# Evolution of Scintillation and Electrical Characteristics of AlGaN Double-Response Sensors During Proton Irradiation

**DOI:** 10.3390/s19153388

**Published:** 2019-08-01

**Authors:** Tomas Ceponis, Kazimieras Badokas, Laimonas Deveikis, Jevgenij Pavlov, Vytautas Rumbauskas, Vitalij Kovalevskij, Sandra Stanionyte, Gintautas Tamulaitis, Eugenijus Gaubas

**Affiliations:** 1 Institute of Photonics and Nanotechnology, Vilnius University, Sauletekio ave. 3, LT-10257 Vilnius, Lithuania; 2 Centre for Physical Sciences and Technology, Sauletekio ave. 3, LT-10257 Vilnius, Lithuania

**Keywords:** GaN, AlGaN, proton induced luminescence, radiation defects, dosimetry, scintillation characteristics, electrical characteristics

## Abstract

Wide bandgap AlGaN is one of the most promising materials for the fabrication of radiation hard, double-response particle detectors for future collider facilities. However, the formation of defects during growth and fabrication of AlGaN-based devices is unavoidable. Furthermore, radiation defects are formed in detector structures during operation at extreme conditions. In this work, study of evolution of the proton-induced luminescence spectra and short-circuit current has been simultaneously performed during 1.6 MeV proton irradiation. GaN and AlGaN (with various Al concentrations) epi-layers grown by metalorganic chemical vapour deposition technique and Schottky diode structures have been examined. Variations of spectral and electrical parameters could be applied for the remote dosimetry of large hadron fluences.

## 1. Introduction

The wide bandgap AlGaN with varied Al content is one of the most promising materials for fabrication of radiation hard [1,2], double-response particle detectors [3] in particle accelerator facilities. However, the formation of extended and point defects during growth and fabrication of AlGaN based devices is unavoidable. This leads to the appearance of ultraviolet (UV) photo-luminescence peaks which are shifted to the short-wavelength range with increase of Al content within this ternary material [4]. The radiation defects are formed in detector structures during operation at extreme fluence conditions which can modify optical and electrical characteristics. Modifications of these characteristics can be applied for dosimetry of high energy radiations. Discovery of the most efficient structures for fabrication of the solar-blind, high sensitivity and wide dynamic range double-response particle detectors and dosimeters is important for design of the advanced devices, capable of operating in harsh environments of high-luminosity particle accelerators.

Study of the evolution of the proton-induced luminescence spectra and diode short-circuit current during 1.6 MeV proton irradiation has been simultaneously performed in this work. GaN and AlGaN (with various Al content) epi-layers grown by metalorganic chemical vapour deposition technique have been examined. Thin epi-layers ensured the penetrative conditions for 1.6 MeV protons. To evaluate a correlation of the parameters of emission and cross-sections of the photon–electron coupling, ascribed to technological and radiation defects in the GaN/AlGaN crystals, the complementary ex situ measurements were performed by pulsed photo-ionization spectroscopy on several GaN/AlGaN samples and by UV pulsed laser-induced photo-luminescence in pristine and the largest proton fluence irradiated samples.

It has been shown in this work that these combined methods of the in situ and ex situ measurements enable ones to trace evolution of the electrical and scintillation characteristics of AlGaN layers with proton fluence up to 4 × 10^15^ cm^−2^. Variations of spectral and electrical parameters could be applied for the remote dosimetry of large hadron fluences.

## 2. Sample Preparation

**Figure 1:**
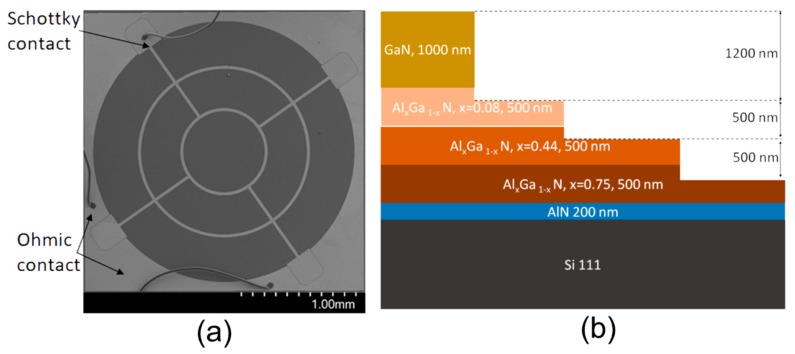
(**a**) The microscopy image of the commercial photo-detectors GUVA–GUVC. Schottky and ohmic contacts are indicated by arrows. (**b**) The diagram of the mesa structure of GaN-AlGaN layers uncovered by reactive ion etching technique.

Several sets of samples were examined. The first set consists of the commercial photo-detectors GUVA-T216D-U, GUVB-T216D-U and GUVC-T206D-U (supplied by Roithner Laser Technik GmbH, Vienna, Austria), adapted for registration of different spectral range radiation. Therefore, these planar Schottky diodes were made of AlGaN materials with different Al content. For spectral measurements during irradiation, the mount of the detector was removed. The microscopy image of the commercial photo-detectors with indicated Schottky and ohmic contacts is shown in Figure 1a.

**Figure 2:**
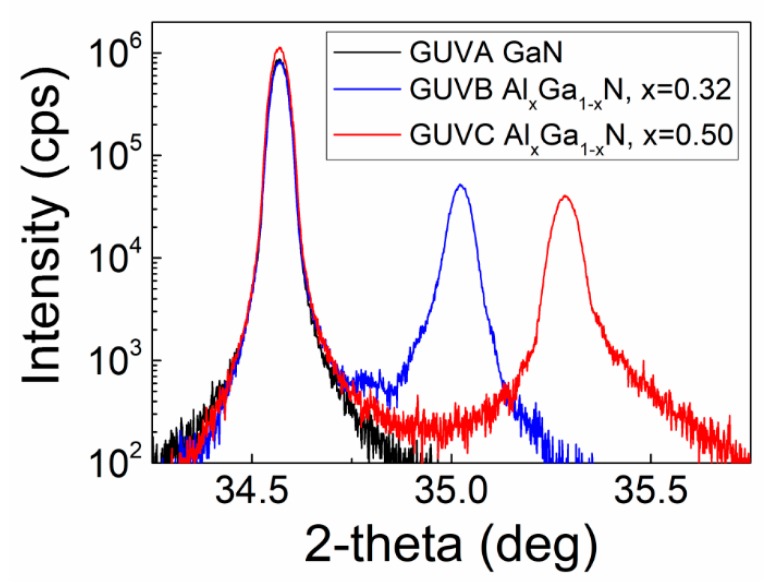
The XRD spectra recorded on GUVA–GUVC samples.

This bare diode structure was examined by X-ray diffraction (XRD) and scanning electron microscopy (SEM) together with energy dispersed X-ray spectroscopy (EDXS) techniques. A SEM instrument Hitachi SU8230 (obtained from Hitachi High-Technologies Corporation, Tokyo, Japan) equipped with an EDX Bruker spectrometer (supplied by Bruker Nano GmbH, Berlin, Germany) was then employed. Samples were XRD characterized using a high resolution (HRXRD) Rigaku SmartLab instrument equipped with Ge(400) double monochromator and scintillation detector SC-70 (all components obtained from Rigaku, Tokyo, Japan). The XRD 2-theta spectra obtained for these GUVA–GUVC samples are illustrated in Figure 2. Sample GUVA contained only GaN layer, while GUVB and GUVC samples showed the AlGaN layers of different Al concentration. Al content has been calculated from difference in 2-theta values using Vergard’s law. Values of the percentage aluminium content of 32% and 50% were thereby determined for the GUVB and GUVC samples, respectively.

The elemental content determined by EDXS for these GUVA–GUVC samples is listed in Table 1.

It can be deduced from Table 1 that samples also contain carbon (C), oxygen (O) and silicon (Si) impurities.

The second set of samples was prepared as mesa structures made of top 1 μm thick GaN layer and several AlGaN layers underneath grown on Si substrates (Figure 1b). The AlGaN material have a 500 nm thick epi-layers of different Al content. These definite AlGaN layers were layer-by-layer uncovered by reactive ion etching (RIE) technique. The samples were placed within a vacuumed chamber of OXFORD Instruments Plasmalab System 100 RIE equipment. The special masks were made to shield etching plasma to form dedicated mesa steps. Chlorine and argon inductively coupled plasma (ICP) was used for etching. The layer thickness and content were verified by the structure profiling and XRD means. The percentage of Al as 8%, 44% and 75% have been determined by XRD for the underneath AlGaN uncovered three mesa steps.

The latter mesa steps were covered by metal mask, impermeable for proton beam, during irradiation, successively leaving only single mesa-step uncovered to have a response only from the single layer. The 1.6 MeV irradiations were performed by a Tandetron 4110A accelerator with proton currents in the range of 20–40 nA. The GUVA–GUVC diode structures also were electrically connected to Keithley pico-ampermeter for simultaneous control of the short-circuit current (*I_SC_*) induced by a proton beam.

## 3. Evolution of Scintillation and Short-Circuit Current in GaN/AlGaN Structures

### 3.1. The Mesa Structure Samples

**Figure 3:**
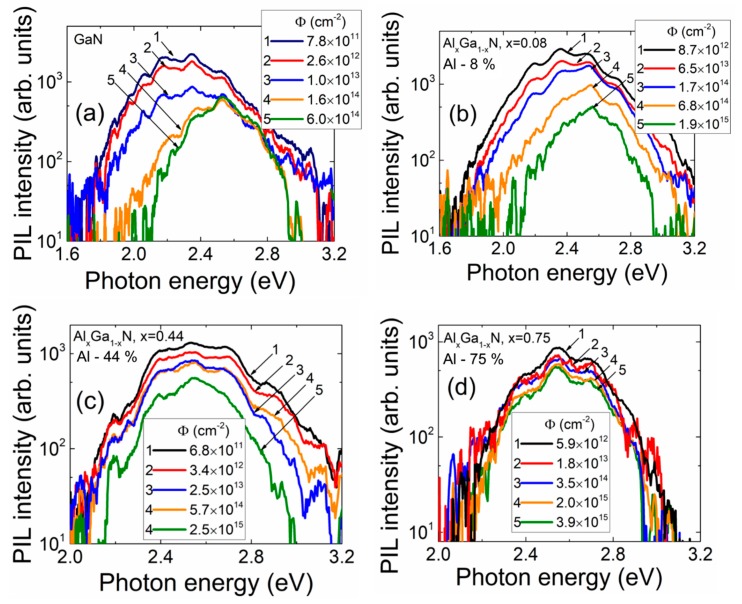
Fluence-dependent evolution of proton-induced scintillation spectra recorded on different Al content (0% (**a**); 8% (**b**); 44% (**c**); 75% (**d**)) mesa structure layers, respectively.

The fluence (*Φ*)-dependent evolution of the proton-induced luminescence (PIL) spectra in different layers of the mesa structure samples with various content of aluminium is illustrated in Figure 3. 

**Figure 4:**
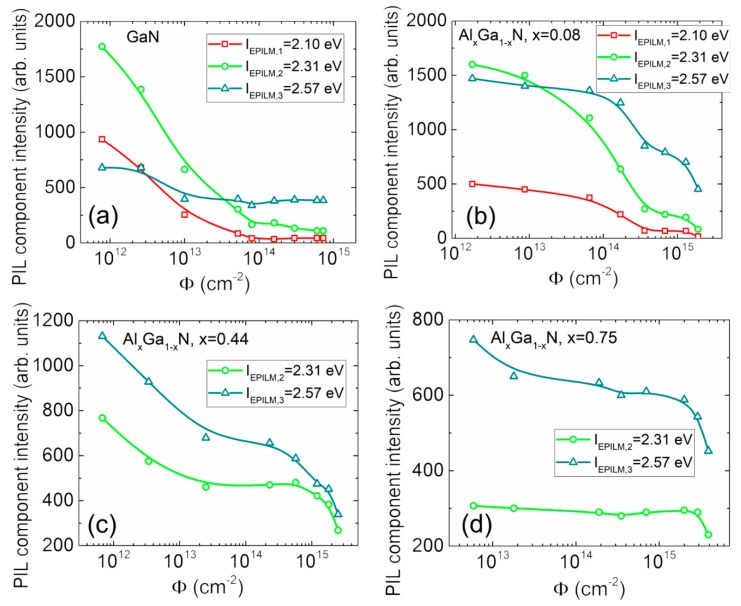
Fluence-dependent variations of the intensity of different spectral components within proton-induced luminescence (PIL) on different Al content (0% (**a**); 8% (**b**); 44% (**c**); 75% (**d**)) mesa structure layers, respectively.

The main feature of this evolution in the set of mesa structure samples is a decrease of the intensity of the long-wavelength component with enhancement of proton irradiation fluence. The decrease of all the spectral components is only observed in GaN and AlGaN epi-layers with the increase of Al content and irradiation fluence (Figure 3a–d). The PIL spectra have been spread into three spectral components for quantitative evaluation of fluence-dependent changes of PIL intensity. The fluence-dependent variation of peak intensity of the separated PIL bands with irradiation fluence are presented in Figure 4 for the mesa structure samples. These dependences imply that the fluence value calibrated PIL intensity reduction can be applied for dosimetry of penetrative particle irradiation with different sensitivity attributed to various PIL spectral bands and content of Al. The steepest decrease appears for the long wavelength components; however, the dynamic range of dosimetry is the narrowest using the latter characteristics. Moreover, for the samples with the largest (75%) content of Al (Figure 3d), the PIL spectrum becomes narrow with small dynamic range of intensity variations in the spectral band peaked at 2.3–2.6 eV.

**Figure 5:**
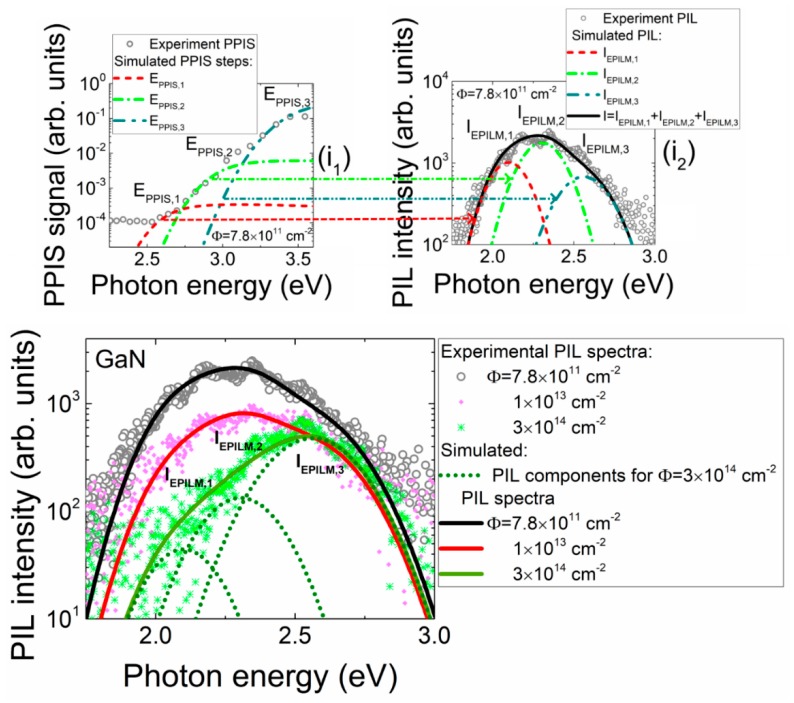
Sketch of association of defects to the predominant spectral peaks within fluence-dependent variations of the intensity of different spectral components within PIL. In the insets: the recorded and simulated PPIS steps (**i**1) and PIL spectral components obtained through simulations of conversion from absorption to emission (**i**2) made for respective PPIS steps.

To identify the origin of different PIL components, the predominant spectral components have been correlated with pulsed photo-ionization spectra (PPIS), measured on satellite mesa structures of the same material. The PPIS technique and its application for defect spectroscopy in GaN materials is described in recent our articles [5,6,7,8]. The recorded and simulated PPIS spectral steps, ascribed to the definite defects responsible for the PIL spectral components, are illustrated within inset 1 for Figure 5. Based on these PPIS components, the luminescence bands (inset 2 for Figure 5) were consequently simulated [5,6] by using the van Roosbroeck–Shockley (vRS) approach [9,10] to correlate the conversion from absorption (PPIS) to Stokes shifted emission (PIL). The latter fitted luminescence components, which peaked at 2.57 eV, 2.31 eV and 2.10 eV, were chosen to deduce an evolution of the PIL spectral components. These PIL spectral components have been ascribed to (C_N_O_N_)^0^ [11] (peaked at ~3.35 eV in PPIS and ~2.57 in PIL), C_N_^-^ [11] (peaked at 2.92 eV in PPIS and 2.31 eV in PIL) and V_Ga_ [11] (peaked at 2.57 eV [12] in PPIS and 2.10 eV in PIL) defects.

The fluence-dependent PIL evolution has been consequently fitted by varying the amplitude only of the chosen (deduced from PPIS and vRS PL analysis as sketched in the insets 1 and 2 for Figure 5) luminescence peaks. The fits of this fluence-dependent PIL evolution are sketched in Figure 5. The fluence-dependent variations of the intensity of different spectral components within PIL were thereby composed from the fits illustrated in Figure 5. The evolution of the intensity of these PIL spectral components can be explained by varied initial filling of the predominant defects due to irradiation. The filling level of various traps is modified by non-radiative recombination centres introduced by proton irradiation.

### 3.2. Schottky Diode Structures

**Figure 6:**
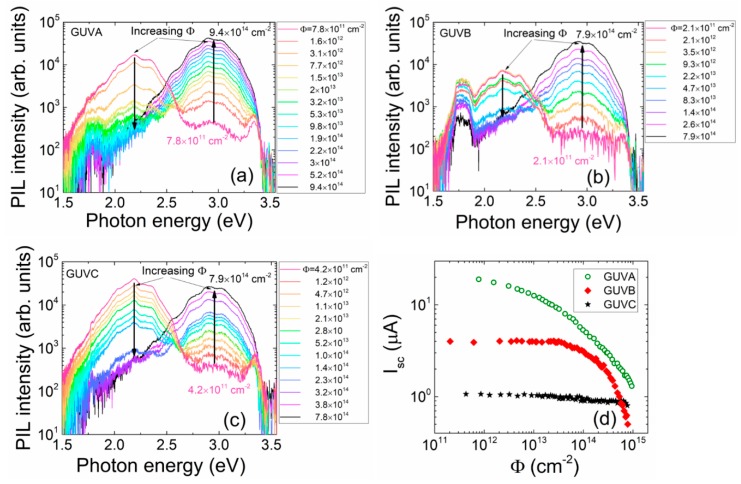
Fluence-dependent evolution of proton-induced scintillation spectra recorded on different Al content (0% (**a**); 32% (**b**); 50% (**c**)) containing diodes GUVA–GUVC, respectively. (**d**) Evolution of SC current in different GUVA–GUVC diodes.

Evolution of PIL spectra recorded on the set of GUV diodes during proton irradiation is illustrated in Figure 6a–c. The general feature of spectra variations is a decrease of intensity of the long wavelength PIL band with further enhancement of the short wavelength band under increase of proton irradiation fluence. However, the rate of fluence-dependent intensity variations is specific for diodes made of different Al content containing materials. The long wavelength band split can be hinted for GaN and Al_0.32_Ga_0.68_N material diodes.

The fluence-dependent short-circuit current (*I_SC_*) variations (Figure 6d), measured simultaneously with PIL spectra, differ significantly for diodes made of different Al content materials. The largest *I_SC_* values have been observed for pure GaN material (GUVA) diodes. Consequently, the dynamic range of *I_SC_* variations is the widest using these GUVA diodes. Thereby, sensitivity to fluence changes is also the highest for the GUVA diodes. An increase of bandgap with Al content leads to reduction of the *I_SC_* current. Therefore, a recordable change of *I_SC_* as a function of fluence is obtained in 32% Al containing material only for the moderate fluences of *Φ* > 3 × 10^13^ p/cm^2^. Even the less values and changes of *I_SC_* (for *Φ* > 10^14^ p/cm^2^) were obtained in diodes (GUVC) of 50% Al content (Figure 6d).

As can be deduced from Figure 6, the spectral and intensity variations of PIL provide the more reliable characteristics in dosimetry detection.

**Figure 7:**
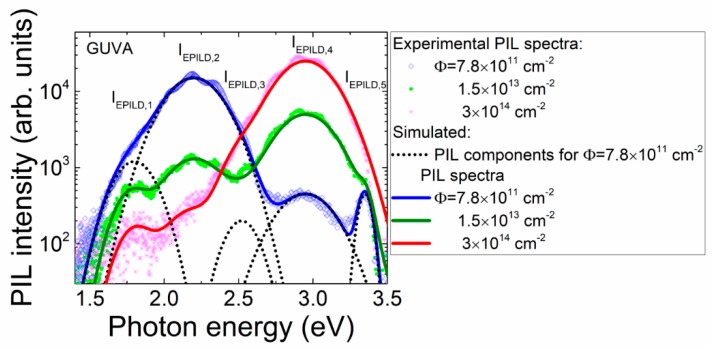
Sketch of deconvolution of the predominant spectral peaks and the fluence-dependent evolution of the intensity of different spectral components within PIL in the GUVA diode.

It appeared that the PIL spectra should be spread into five spectral components for GUV diode structures to quantitatively reproduce the fluence-dependent changes of PIL spectrum and intensity. The Gaussian approximation of the spectral component shape *S (hν, σ) = A_f_exp*[*−(hν − E_f_)*^2^*/σ*^2^] with *A_f_* as the peak intensity of the *f-*th component with peak energy *E_f_* and *σ* the band width has been accepted in fitting of PIL spectra. A sketch of the deconvolution of the predominant spectral peaks and the fluence-dependent evolution of the intensity of different spectral components within PIL in the diode GUVA is presented in Figure 7. There, symbols denote the experimental data, thick solid lines represent a total (sum) of intensities of the separate components shown by thin dotted lines, simulated for spectrum obtained at fixed irradiation fluence. In simulations of the total PIL intensity as a function of the photon energy *hν*, only the peak intensity *A_f_* of definite spectral components was modified as a free variable. In addition to PIL spectral bands peaked at 2.10 eV, 2.31 eV as well as 2.57 eV in mesa structures, the PIL peaks at 1.8 eV, 2.52 eV, 2.95 eV and 3.35 eV appear in GUV diodes. This implies that point defect structure slightly differs for mesa and diode samples. The 2.52 eV and 2.95 eV peak intensities increase with fluence indicating the main difference of AlGaN structures grown on Si and commercially manufactured materials. It seems that RIE etching does not produce additional defects, as GUV diodes PIL spectra are richer than that of mesa structures.

**Figure 8:**
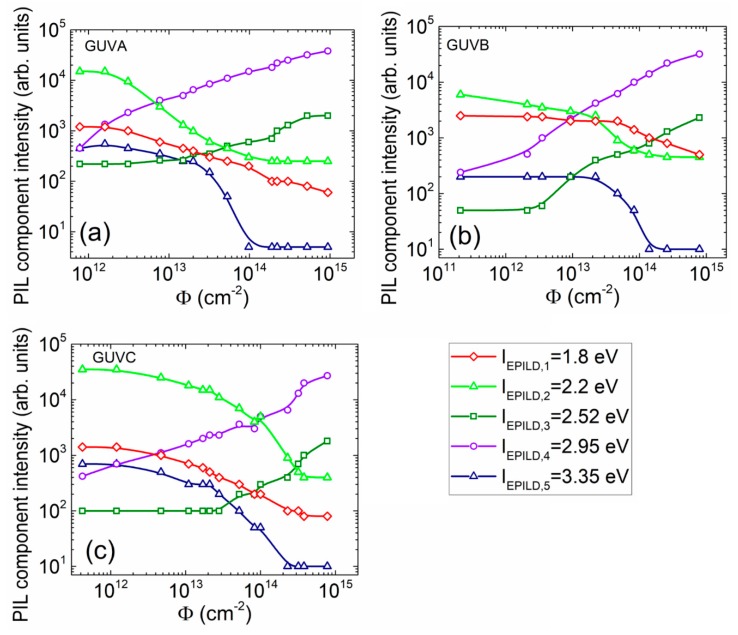
Fluence-dependent variations of the intensity of different spectral components within PIL obtained for different Al content (0% (**a**); 32% (**b**); 50 % (**c**)) GUV diodes, respectively.

The fluence-dependent peak intensities, extracted from simulations performed according to the method sketched in Figure 7, and attributed to different spectral components, are plotted in Figure 8. It can be deduced from Figure 8 that the largest intensities and the widest dynamic range are obtained for the *E_PILD,2_* and *E_PILD,4_* spectral components in all the examined diodes. Thereby, these components can be employed for dosimetry based on PIL spectral variations, as the largest intensity determines the highest sensitivity and reliability of spectrum recording.

The spectral components have been tentatively associated with defects in ternary AlGaN materials identified in literature referenced. These components are listed in Table 2. The peak energy values ascribed to definite spectral components (inherent for the full range of Al content (*x*) possible changes) are there denoted and ascribed to the specific defects in Table 2.

Thereby, the Ga vacancies or DAP centres and V-O complexes prevail in both sets of AlGaN sample sets, keeping in mind the blue-shift of PIL peak position with enhancement of *x* ascribed to definite defects.

## 4. Discussion

The stability of the fluence-dependent modifications of the spectral and electrical characteristics has been verified by comparing the UV pulsed laser-excited photo-luminescence (PL) spectra and electrical signals measured ex situ before and after proton irradiations. The coincidence of spectrum structures and *I_SC_* values have been obtained. Also, the concentration of the proton generated secondary electron–hole pairs have been estimated by comparing the intensity of spectral PL components and *I_SC_*. These measurements are inevitable in calibration of the double-response dosimeters.

It can be deduced from Figure 4, Figure 6d and Figure 8 that the most acceptable structures in design of the double-response dosimeters are GaN/Al_0.08_Ga_0.92_N structures. Concerning the aspect of linearity of the PIL dose-dependent characteristic, control of the PIL spectral component *E*_PILD,4_ peaked at 2.95 eV would be preferable (Figure 8). The AlGaN epi-layers of enhanced thickness ≥1 μm (when comparing GUVA diodes and mesa structures) are preferable to have the relevant interaction depth for the penetrative particle irradiations. To simultaneously record the most reliable optical and electrical responses, the Ga vacancy and oxygen-rich AlGaN materials with small content of Al and doped with carbon would be desirable in fabrication of the solar-blind and wide dynamic range dosimeters. The point defects prevail in formation and dose-dependent modification of PIL spectra regardless of high density of dislocations, inherent for the MOCVD-grown AlGaN materials. The junction structures are preferable to get the electrical signals. The different type electrical signals as *I_SC_*, barrier capacitance [3], etc. can then be employed for the in situ control of the particle beam induced changes of recorded characteristics.

**Figure 9:**
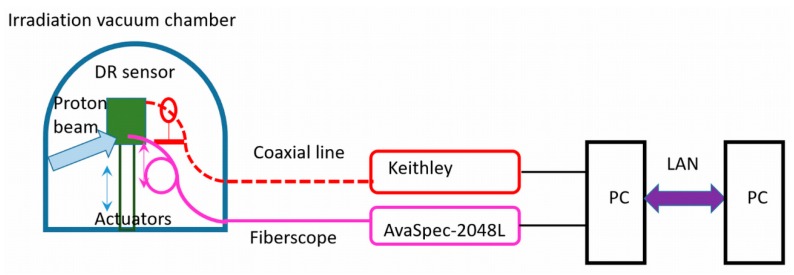
A scheme of remote dosimetry using double-response (DR) AlGaN sensor.

The AlGaN structures can be applied for the remote sensing of particle beams and of accumulated their fluences. The solar-blind and small leakage current AlGaN detectors are insensitive to the visible (VIS)–near infrared (NIR) spectrum background emissions and thereby preferable in comparison with Si-based sensors. A tentative scheme (employed in this work) for remote dosimetry of particle beams is sketched in Figure 9, which allows to perform measurements in vacuum chamber and harsh environment of irradiations. The vacuum-proof electrical and fiber connections have been there exploited for output of the electrical and PIL signals. The multi-string UV clear (up to 240 nm) fiberscopes (BF20HSMA01—Round Fiber Bundle, V2H6S—Fiber Feedthrough as well as M93L02—Fiber Patch Cable (supplied by Thorlabs, Inc., Newton, New Jersey, United States)) and an AvaSpec-2048L spectrophotometer (obtained from Avantes BV, Apeldoorn, Netherlands) were employed to transfer and record the PIL signals. The *I_SC_* current was measured by using a Keithley pico-ampermeter. Collection and primary processing of the *I_SC_* and PIL signals as well as proton beam current were performed by personal computer (PC). Control of regimes of the measurement devices and data display was implemented via LAN connection on the remote PC.

## 5. Summary

The AlGaN structures of different Al content have been examined in order to fabricate the efficient double-response particle sensors and dosimeters. The double-response sensors are preferable when it is important to simultaneously control the instantaneous particle flux and the accumulated fluence at the fixed location of the sensor or to increase sensitivity and reliability of the dosimeter in the range of large fluences. It has been shown that the most acceptable structures in design of the double-response dosimeters are GaN/Al_0.08_Ga_0.92_N structures. The linearity of the PIL dose-dependent characteristic can be achieved for the PIL spectral component peaked at 2.95 eV. The defects prevailing in modifications of PIL spectra have been tentatively identified. The point defects are predominant in formation and dose-dependent modification of PIL spectra regardless of the high density of dislocations inherent for the MOCVD-grown AlGaN materials. The Ga vacancy and oxygen-rich AlGaN materials with small content of Al and doped with carbon would be desirable in the fabrication of wide dynamic range dosimeters. The junction structures are preferable to record the different types of electrical signals. A possible scheme for remote dosimetry of particle beams has been approved in this work for measurements in a vacuum chamber and harsh environment of irradiations.

## Figures and Tables

**Table 1:** Elemental content in GUVA–GUVC samples determined by EDXS.

Diode	C	N	O	Al	Si	Ga
GUVA	9.08	24.39	23.04	0.15	5.53	37.10
GUVB	7.75	23.81	31.32	6.90	6.72	22.87
GUVC	6.05	19.79	33.70	11.01	8.10	20.84

**Table 2:** Association of the luminescence spectral peaks to different point defects in GaN-AlGaN materials according to literature data.

Al_x_Ga_1−x_N		
PIL Peak Quantum Energy (eV)	Defect Type	Reference
E_PILD,1_ = 1.27 for *x* = 0; 1.89 for *x* = 1; 1.8 for *x* = 0; 1.9 for *x* = 0	(V_III_-O_N_)^2−/1−^; V_Ga_ related complex; shallow donor and a deep acceptor pair	[13] [14] [15]
E_PILD,2_ = 2.15 for *x* = 0; 3.9 for *x* = 1	V_Ga_ or V_Ga_ related complex	[16]
E_PIDL,3_ = 2.20 for *x* = 0; 3.9 for *x*~1	V_Ga_-O_N_; shallow donor and a deep acceptor pair; V_III_-O_N_	[17,18] [19] [13]
E_PILD,4_ = 2.80 for *x* = 0; 3.0	V_III_-O_N_ related	[20]
E_PILD,5_ = 2.80 for *x* = 0; 3.17	V_III_ related	[21]
